# Hypoxia-Related Hormonal Appetite Modulation in Humans during Rest and Exercise: Mini Review

**DOI:** 10.3389/fphys.2017.00366

**Published:** 2017-05-30

**Authors:** Tadej Debevec

**Affiliations:** Department of Automation, Biocybernetics and Robotics, Jozef Stefan InstituteLjubljana, Slovenia

**Keywords:** altitude, hypoxemia, satiety, hunger, regulation

## Abstract

Obesity is associated with numerous chronic ailments and represents one of the major health and economic issues in the modernized societies. Accordingly, there is an obvious need for novel treatment approaches. Recently, based on the reports of reduced appetite and subsequent weight loss following high-altitude sojourns, exposure to hypoxia has been proposed as a viable weight-reduction strategy. While altitude-related appetite modulation is complex and not entirely clear, hypoxia-induced alterations in hormonal appetite modulation might be among the key underlying mechanisms. The present paper summarizes the up-to-date research on hypoxia/altitude-induced changes in the gut and adipose tissue derived peptides related to appetite regulation. Orexigenic hormone ghrelin and anorexigenic peptides leptin, glucagon-like peptide-1, peptide YY, and cholecystokinin have to-date been investigated as potential modulators of hypoxia-driven appetite alterations. Current evidence suggests that hypoxia can, especially acutely, lead to decreased appetite, most probably via reduction of acylated ghrelin concentration. Hypoxia-related short and long-term changes in other hormonal markers are more unclear although hypoxia seems to importantly modulate leptin levels, especially following prolonged hypoxic exposures. Limited evidence also suggests that different activity levels during exposures to hypoxia do not additively affect hormonal appetite markers. Although very few studies have been performed in obese/overweight individuals, the available data indicate that hypoxia/altitude exposures do not seem to differentially affect appetite regulation via hormonal pathways in this cohort. Given the lack of experimental data, future well-controlled acute and prolonged studies are warranted to expand our understanding of hypoxia-induced hormonal appetite modulation and its kinetics in health and disease.

## Introduction

Obesity represents one of the key health issues in many western societies and is associated with numerous chronic ailments (Ng et al., 2014). Given that, over one billion of world population is currently overweight and at least 300 million people obese, there is an obvious need for new therapeutic strategies (Ng et al., 2014). Sojourns to high altitude and/or exposures to hypoxia have recently been proposed as a potential novel weight-loss strategy (Netzer et al., 2008; Quintero et al., 2010; Kayser and Verges, 2013). The rationale is based on anecdotal as well as scientific observations of significant body mass decreases following high altitude sojourns. Reductions in body mass observed at high altitudes seem to be a consequence of blunted appetite resulting in decreased energy intakes (Westerterp and Kayser, 2006; Benso et al., 2007; Kalson et al., 2010). This phenomenon, also termed “Altitude anorexia” was initially thought to be associated with altitude-related environmental factors (cold, dehydration, etc.) and medical conditions (acute mountain sickness) (Hackett and Roach, 2001). However, growing body of literature from well-controlled laboratory investigations indicates that hypoxia *per se* underlies the observed appetite changes (Westerterp-Plantenga et al., 1999; Wasse et al., 2012; Bailey et al., 2015).

While hypoxia-related appetite modulation is not completely understood, alterations in hormonal appetite regulation might be one of the key underlying mechanisms (Quintero et al., 2010; Kayser and Verges, 2013). The purpose of the present paper is to summarize to-date research on hypoxia/altitude-induced changes in the gut and adipose tissue derived peptides related to appetite regulation. Contemporary studies suggest that orexigenic hormone ghrelin and anorexigenic agents leptin, glucagon-like peptide-1 (GLP-1), peptide YY (PYY), and cholecystokinin (CCK) are among the main potential hormonal modulators of hypoxia-driven appetite changes. In addition to reviewing the current evidence regarding these hormonal markers, the review will also address the potential influence of different activity levels on hormonal appetite regulation under hypoxic conditions. Finally, considerations regarding future research directions are also discussed.

## Hormonal appetite regulation

Energy balance is dynamically maintained by a complex integration of afferent and efferent metabolic and neural signals orchestrated by the central nervous system. It is indeed fascinating that most mammals are capable of maintaining relatively stable body mass over their lifespan despite significant fluctuations in energy expenditure on the one hand, and energy intake on the other (Strader and Woods, 2005). This indicates that long-term appetite control is tightly controlled and closely related to energy expenditure. Hypothalamus plays one of the main roles in the central appetite control (Schwartz, 2006). In particular, the efferent signals from the gut, pancreas, liver, and adipose tissue get integrated within the hypothalamus which then modulates appetite in order to maintain energy homeostasis (Hussain and Bloom, 2013). Several hormonal markers are involved in both, long (days-years) and short-term (meal-to-meal) appetite regulation. Leptin is one of the crucial peptides modulating long-term energy balance and constantly provides tonic signals from the body fat stores (Strader and Woods, 2005). On the other hand, various peptides, originating mostly from the gastrointestinal tract, modulate appetite in the short-term. These peptides signal meal-to-meal fluctuations in immediate energy availability and thereby regulate short-term changes in satiety and hunger control (Perry and Wang, 2012). While the key hormonal markers are briefly summarized below, the interested readers are referred to a number of comprehensive reviews on the topic (Neary et al., 2004; Strader and Woods, 2005; Huda et al., 2006; Coll et al., 2007; Stensel, 2010; Perry and Wang, 2012).

As mentioned above leptin is the key long-term satiety signaling peptide produced almost exclusively within the adipocytes (Zhang et al., 1994). The systemic circulating leptin levels closely mirror the amount of adipose tissue (Shimizu et al., 1997) and tend to decrease and increase in response to starvation and overfeeding, respectively. While the critical importance of leptin in maintaining energy balance through direct anorexigenic signaling to the hypothalamus is clearly established, it is of note that the ability of leptin to inhibit appetite can be blunted (Enriori et al., 2006). This phenomenon, also termed “leptin resistance” is commonly observed in obese and overweight individuals. Another adipose tissue derived peptide proposed to play important role in energy balance is adiponectin (Trujillo and Scherer, 2005). In contrast to leptin, adiponectin concentration is inversely related to adipose tissue mass and seems to augment appetite via adenosine monophosphate-activated protein kinase stimulation within the hypothalamus (Steinberg and Kemp, 2007).

Peptide GLP-1 has been shown to promote satiety through its direct effect on central appetite regulation (Naslund et al., 1999) and also indirectly by reducing gastric emptying and suppressing gastric acid secretion (Verdich et al., 2001). GLP-1 is released from the endocrine L-cells within the small and large intestine in response to feeding and is one of the most powerful incretin hormones (Drucker, 2006), significantly influencing insulin secretion and biosynthesis within the pancreas (Baggio and Drucker, 2007). It is of note, that peripherally the GLP-1 exists in its active (GLP-1_7–36_) and inactive (GLP-1_9–37_) form. Concurrently with the GLP-1, the endocrine L-cells of the intestines release the PYY which is also a potent short-term appetite modulating peptide with an anorexogenic effect (Chaudhri et al., 2006). While the circulating PYY levels in fasting state are low they increase rapidly in response to food intake when PYY_1–36_ and PYY_3–36_ forms are released into the circulation, with the latter being the most abundant and active form. PYY mostly mediates its appetite effects through neuropeptide Y receptors and is additionally involved in gastric motility inhibition and electrolyte absorption augmentation in the gut (Batterham and Bloom, 2003). Another gut-derived peptide implicated in satiety signaling is CCK. It was the first discovered gut peptide demonstrated to be an important modulator of postprandial satiety (Kissileff et al., 1981). The CCK is released in the circulation, in response to fatty acids, from the I cells of the small intestine and exerts its action via the influence of CCK1 receptor on the hypothalamus and the brainstem (Moran et al., 1992). Besides its appetite-related effects, the CCK also promotes fat and protein digestion (Liddle et al., 1985).

Ghrelin, also known as the “hunger hormone,” is an enteric peptide involved in short-term energy balance maintenance. Interestingly, it is the only orexigenic appetite-related gut signal discovered to date (Williams and Cummings, 2005) and is produced exclusively within the oxyntic glands of the stomach. It is well established that ghrelin levels decrease postprandially and are increased prior to meals (Cummings et al., 2001). Augmented ghrelin levels promote gastric motility, growth hormone release and attenuate fat utilization (Kojima and Kangawa, 2005). It is important to note that ghrelin tends to exert the orexigenic effect only in its acylated form (Ghigo et al., 2005). Given that the acylated ghrelin form represents only 10–20% of the total circulating ghrelin, this notion needs to be taken into account when interpreting the outcomes of the studies investigating its appetite-related function.

Obviously, there are also other peptides that have been implicated in the general energy balance maintenance. In particular, the pancreas derived amylin (Roth, 2013) and pancreatic polypeptide (Batterham et al., 2003), interleukins from the adipose tissue (Zorrilla et al., 2007) as well as the oxyntomodulin (Cohen et al., 2003), obestatin (Beasley et al., 2009), and insulin-like peptide 5 (Grosse et al., 2014) produced within the gut have all been shown to influence appetite in a complex interplay with the other incretin and previously discussed hormones. However, given the limited scope, the present paper is focused solely on the appetite-signaling peptides that have already been investigated in regards to their role in hypoxia-related appetite modulation. The main findings of the up-to-date studies on the effects of altitude/hypoxia on these select markers is summarized in the following section.

## Hypoxia-related changes in hormonal regulation

As noted above, appetite reductions are consistently reported in individuals acutely exposed to higher terrestrial altitudes (Westerterp-Plantenga et al., 1999; Westerterp and Kayser, 2006). While this was initially thought to be a consequence of other altitude-related factors (Hackett and Roach, 2001), growing body of literature indicates that altitude-related hypoxia seems to be the main driver of the observed appetite alterations. Indeed, hypoxia has been shown to affect a number of hormonal markers involved in appetite regulation. The outcomes of the key well-controlled short-term and long-term investigations on the effects of hypobaric (terrestrial) and/or normobaric (simulated altitude) hypoxia are presented in Tables 1, 2, respectively. Within the present review, the term acute (short-term) and prolonged (long-term) exposures are used to define hypoxic exposures of ≤ 24-h and >24-h duration, respectively.

**Table 1:** Key findings from the controlled studies investigating the effects of acute and short-term hypoxic/altitude exposures on select orexigenic and anorexigenic hormonal markers.

**Study Outline**	**Participants**	**Orexigenic markers**	**Anorexogenic markers**	**Conclusions**	**Reference**
17-h NH (F_i_O_2_ = 12.4%)	Healthy, untrained individuals (*N* = 25)	–	↑ leptin	Nocturnal hypoxia increases leptin, but not GLP-1. Changes in leptin were correlated to S_p_O_2_ alterations.	Snyder et al., 2008
Pre–Post			↔ GLP-1		
7-h NH (F_i_O_2_ = 12.7%)	Healthy, untrained individuals (*N* = 10)	↓ acylated ghrelin	↔ total PYY	Acute hypoxia suppresses acylated ghrelin concentration while only a tendency for a decrease was noted in total PYY.	Wasse et al., 2012
4 conditions (cross-over):					
normoxia, hypoxia, normoxic exercise, hypoxic exercise					
Pre–Post					
1-day HH @ 3,454 m	Healthy, untrained individuals (*N* = 11)	↓ total ghrelin ↓ postprandial total ghrelin	↓ CCK (14 h)	Acute hypobaric hypoxia induces significant reduction in fasting and postprandial total ghrelin and CCK.	Riepl et al., 2012
Pre–14 h–24-h			↓ postprandial CCK (24 h)		
24-h NH (F_i_O_2_ = 13.9%)	Healthy, untrained individuals (Hypoxia *N* = 8) (H. exercise *N* = 6)	↔ total ghrelin	↔ leptin ↔ total PYY	Acute normobaric hypoxia does not significantly alter hormonal appetite regulation. No additive effect of exercise.	Debevec et al., 2014
2 groups: hypoxia,			↔ GLP-1		
hypoxic exercise,			↔ Adiponectin		
Pre–Post					
2.6-h NH (F_i_O_2_ = 12.7%)	Healthy, untrained individuals (*N* = 12)	↓ acylated ghrelin	↔ total PYY	Acute normobaric hypoxia suppresses appetite and acylated ghrelin concentrations.	Bailey et al., 2015
4 conditions (cross-over):			↔ GLP-1		
moderate and high intensity exercise in normoxia and hypoxia				No influence of exercise intensity was noted.	
Pre–Post					
7-h NH (F_i_O_2_ = 15.0%)	Healthy, untrained individuals (*N* = 8)	↔ total ghrelin	↔ leptin	Seven-hour exposure to moderate hypoxia does not alter appetite related hormonal markers nor perceived appetite.	Morishima and Goto, 2016
2 conditions (cross-over):			↔ GLP-1		
hypoxia, normoxia					

*HH, Hypobaric hypoxia; NH, Normobaric hypoxia; FiO2, Fraction of inspired O2; SpO2, Capillary oxygen saturation; Pre, testing before the exposure; Post, testing just before cessation of the exposure GLP-1, glucagon-like peptide 1; PYY, Peptide YY; CCK, Cholecystokinin; ↓, significantly decreased; ↑, significantly increased; ↔, no significant changes*.

**Table 2:** Key findings from the controlled studies investigating the effects of long-term hypoxic/altitude exposures on select orexigenic and anorexigenic hormonal markers.

**Study Outline**	**Participants**	**Orexigenic markers**	**Anorexogenic markers**	**Conclusions**	**Reference**
12-day HH @ 5,050 m Pre–Day 1–Day 12	Healthy, active individuals (*N* = 12)	–	↓ leptin (Day 1) ↓ leptin (Day 12)	Exposure to high altitude reduces leptin levels. Leptin reduction was not associated with AMS.	Zaccaria et al., 2004
7-day HH @ 4,300 m Pre–Day 2–Day 7	Non-acclimatized (*N* = 30) & acclimatized (*N* = 50) soldiers	↓ total ghrelin (Day 2) ↔ total ghrelin (Day 7) ↑ total ghrelin in accl. vs. non-acclimatized.	↑ leptin (Day 2) ↑ leptin (Day 7) ↑ leptin in accl. vs. non-acclimatized	High altitude exposure augments leptin and reduces total ghrelin concentrations.	Shukla et al., 2005
7-day HH @ 2650 m Pre–Post	Obese individuals with metabolic syndrome (*N* = 20)	↔ total ghrelin	↑ leptin	Despite significant body mass reductions, leptin was higher and ghrelin was unchanged following moderate altitude exposure.	Lippl et al., 2010
4-day HH @ 4559 m Pre–Day 2–Day 4	Healthy, experienced mountaineers (*N* = 32)	–	↔ total PYY (Day 2 & 4) ↔ CCK (Day 2 & 4)	None of the measured hormones was significantly altered in response to altitude.	Aeberli et al., 2013
10-day NH (F_i_O_2_ = 13.9%) 2 groups: hypoxia, hypoxic exercise, Pre–Post	Healthy, untrained individuals (Hypoxia *N* = 8) (H. exercise *N* = 6)	↔ total ghrelin	↔ leptin ↔ total PYY ↔ GLP-1 ↔ Adiponectin	Prolonged exposure to normobaric hypoxia with or without exercise training does not seem to alter hormonal appetite regulation.	Debevec et al., 2014
10-day NH (F_i_O_2_≈ 15%) Cross-over control: Normoxic confinement Pre–Post	Healthy, trained individuals (*N* = 11)	↔ total ghrelin	↑ leptin (both hypoxia & normoxia) ↔ total PYY ↑ GLP-1 (normoxia only)	Ten day confinement to hypoxia and normoxia exerts similar responses in hormonal appetite markers.	Mekjavic et al., 2016
16-day NH (F_i_O_2_ = 14.1%) 3 conditions (Cross-over): Normoxic inactivity, Hypoxic inactivity, Hypoxic activity Pre–Post	Healthy, untrained individuals (*N* = 11)	↔ total ghrelin	↓ leptin (Hypoxia + activity only) ↔ total PYY ↓ GLP-1 (Hypoxia + inactivity only)	The measured hormonal appetite markers indicate hypoxia-related appetite stimulation, although this did not reflect in increased intakes.	Debevec et al., 2016

*HH, Hypobaric hypoxia; NH, Normobaric hypoxia; F_i_O_2_, Fraction of inspired O_2_; AMS, Acute mountain sickness; Pre, testing before the exposure; Post, testing just before cessation of the exposure; GLP-1, glucagon-like peptide 1; PYY, Peptide YY; CCK, Cholecystokinin; ↓, significantly decreased; ↑, significantly increased; ↔, no significant changes*.

Leptin has been one of the first hormones implicated in altitude-related anorexia (Tschop et al., 1998). Mechanistically, hypoxia-induced stimulation of transcription factor HIF-1α, a key regulator of cellular responses to reduced O_2_ availability, can also effect circulating leptin via HIF-1α-dependent expression of the leptin gene expression (Grosfeld et al., 2002). However, albeit the fact that both acute (Tschop et al., 1998; Snyder et al., 2008) and prolonged (Shukla et al., 2005; Lippl et al., 2010; Mekjavic et al., 2016) exposures to hypoxia have demonstrated increases in circulating leptin levels its role in hypoxia-related appetite modulation is unclear. In particular, numerous studies have also demonstrated unaltered (Benso et al., 2007; Debevec et al., 2014; Morishima and Goto, 2016) or even decreased (Zaccaria et al., 2004; Debevec et al., 2016) leptin levels in response to hypoxic exposure. Interestingly, the only to-date study investigating the effects of altitude exposure on leptin levels in obese individuals showed a significant increase following 7-day exposure to 2,650 m (Lippl et al., 2010). It is important to note that the outcomes of field, or not suitably controlled, studies might have been confounded by factors such as environmental influences (i.e., temperature, humidity), diet, activity levels, as well as changes in body composition, all known to importantly modulate leptin release (Sierra-Johnson et al., 2008). In addition, the pulsatile manner of leptin secretion and its circadian variation (Park and Ahima, 2015) might have also explain some of the discrepancies. However, even when taking into account only the outcomes of the laboratory based, strictly-controlled studies, both short (Snyder et al., 2008; Morishima and Goto, 2016) and long-term (Debevec et al., 2014, 2016) exposures led to contrasting leptin responses. Although the effect of hypoxia on leptin release, as well as its influence on hypoxia-related appetite modulation, needs to be clarified it seems that hypoxia does influence leptin concentration, at least in response to long-term exposures.

To-date, hypoxia-related GLP-1 modulation received little attention. Snyder et al. (2008) were the first to assess the influence of acute (17-h) exposure to simulated hypoxia on fasting GLP-1 levels and did not find any independent hypoxic effect. These finding were further corroborated by subsequent acute (Bailey et al., 2015; Morishima and Goto, 2016) and also prolonged (Debevec et al., 2014; Mekjavic et al., 2016) hypoxic exposures. All of these studies did not show any significant effect of different hypoxia levels (fraction of inspired O_2_ (F_i_O_2_) from 12 to 15%) on fasting as well as postprandial GLP-1 levels. Interestingly, postprandial GLP-1 plasma concentration was shown to decrease following 16 days of hypoxic confinement when combined with bed rest-induced inactivity (Debevec et al., 2016). While this might suggest an effect of different activity levels on hypoxia-related GLP-1 modulation, previous two studies on the topic did not elucidate any significant influence of exercise (Debevec et al., 2014; Bailey et al., 2015). Based on the current studies, GLP-1 does not seem to be particularly influenced by environmental hypoxia and thus its potential role and contribution to the complex altitude-related appetite reduction is questionable.

In contrast to GLP-1, a tendency for a decrease in PYY was noted following acute (7-h) hypoxic exposure (Wasse et al., 2012). The study by Wasse et al. (2012) was the first to assess the potential contribution of PYY to hypoxia-provoked appetite changes. Few recent studies also investigated the hypoxia-induced alterations in PYY. However, all of them failed to demonstrate any significant effect of hypoxia *per se* either following acute (Bailey et al., 2015) and prolonged (Aeberli et al., 2013; Debevec et al., 2014, 2016; Mekjavic et al., 2016) hypoxic exposures. The fact that the changes in total PYY instead of its active and most potent form (PYY_3–36_) were assessed in all of the above investigations might underlie the lack of changes. Nevertheless, based on the available data the PYY does not seem to be affected by hypoxia and thus, might not play a role in altitude-related appetite modulation.

Bailey et al. (2000) initially demonstrated that high altitude trekking (up to ~5,100 m) can augment CCK levels in lowlanders and suggested that this increase might underlie the observed food intake reduction. Interestingly they also noted higher CCK responses in those suffering from AMS symptoms than those without. Albeit this initial findings, subsequent study from the same group conversely suggested that hypoxia might reduce CCK levels if applied during acute exercise (Bailey et al., 2001). This is congruent with the only two other remaining studies on the topic suggesting that acute exposure to ~4,500 m does not alter CCK levels (Aeberli et al., 2013) and furthermore that fasting and postprandial CCK levels might be reduced as a consequence of 24-h exposure to ~3,500 m (Riepl et al., 2012). Regardless of the fact that, except for the study by Bailey et al. (2001), all investigations were performed in the field scenarios and might therefore be confounded by other terrestrial altitude-related environmental factors, current evidence does not support the notion that CCK plays an important role in hypoxia-related appetite modulation.

Ghrelin is the only orexigenic gastrointestinal peptide that was implicated in hypoxia-related appetite regulation. More than a decade ago, Shukla et al. (2005) initially demonstrated that acute exposure to terrestrial altitude (~4,300 m) reduces fasting ghrelin although this decrease was not observed following 7 days of altitude residence. Although the exact mechanism of hypoxia-related changes in ghrelin is unclear, the reduction of the liver blood flow, due to reduced O_2_ availability-induced blood redistribution, and subsequent reduction in ghrelin acylation might play a role (Bailey et al., 2015). Subsequent terrestrial investigations provided further support for the altitude-induced acute ghrelin reduction (Riepl et al., 2012) as well as for the lack of changes following long-term altitude residence (Benso et al., 2007). Two recent well-controlled studies performed in normobaric hypoxia further showed that hypoxia *per se* can significantly blunt circulating acylated ghrelin concentration regardless of activity levels (Wasse et al., 2012; Bailey et al., 2015). In contrast, total ghrelin concentration did not seem to change in response to seven (Morishima and Goto, 2016) or twenty-four (Debevec et al., 2014) hour exposures to moderate (F_i_O_2_ = 15.0%) or high (F_i_O_2_ = 13.9%) simulated altitudes, respectively. As suggested by Bailey et al, the discrepancies in the acute findings are most probably a consequence of measuring total in some and acylated ghrelin form in the other studies. Regardless of the above, studies indicate that ghrelin levels do not seem to be reduced following prolonged exposures to normobaric hypoxia (Debevec et al., 2014, 2016; Mekjavic et al., 2016). No changes in total ghrelin were also observed in obese individuals following 7-day exposure to 2,600 m (Lippl et al., 2010). Taken together, hypoxia seems to, at least acutely, blunt ghrelin levels and thereby directly induce appetite reduction. However, the kinetics and long-term hypoxia-induced ghrelin modulation remains currently ambiguous.

## Hypoxia, exercise, and hormonal appetite regulation

Very few studies to date examined the combined effects of hypoxia and exercise on hormonal appetite regulation although this approach seems promising for obesity treatment (Netzer et al., 2008; Urdampilleta et al., 2012; Millet et al., 2016). Besides the already mentioned acute investigations (Bailey et al., 2001; Wasse et al., 2012), only two studies to date scrutinized the influence of concomitant hypoxia and exercise training (4 weeks, three session per week) on select hormonal appetite markers (Haufe et al., 2008; Morishima et al., 2014). Haufe et al. (2008) did not find any additional effect of hypoxia following 4-week training period on both leptin and adiponectin concentrations although their data suggests that hypoxic as compared to normoxic training can induce superior overall metabolic adaptations. Similarly, Morishima and Goto (2016) did not elucidate any hypoxia-dependent effect on fasting or postprandial total ghrelin or leptin concentrations, while the GLP-1 concentration tended to be lower following hypoxic as compared to normoxic training. Also, no additional effect of daily moderate intensity exercise training during 10-day hypoxic confinement was observed on postprandial total ghrelin, PYY and GLP-1 concentrations (Debevec et al., 2014). Collectively, the data from acute and prolonged investigations combining hypoxia and activity do not provide support for importance of activity levels in hypoxia-related hormonal appetite modulation.

## Conclusions

The reviewed data, pertinent to altitude/hypoxia-related hormonal appetite regulation suggests that acutely, exposure to hypoxia can reduce acylated ghrelin concentration and thereby decrease appetite. Hypoxia-related short and long-term changes in other hormonal markers are more unclear although hypoxia seems to importantly modulate leptin levels, especially following prolonged hypoxic exposures. The lack of any significant effects of hypoxia on both total PYY and GLP-1 might suggest that they are not crucial in hypoxia-related appetite regulation. Limited investigations regarding the combined effects of hypoxia and exercise also indicate that different activity levels during hypoxic exposures, do not seem to additively affect hormonal appetite markers.

Given the complexity of hormonal appetite regulation (Coll et al., 2007) and the potential influence of other (environmental) factors, future studies should aim at appropriate standardization of the procedures and strict control of potential confounding factors. This is especially important for long-term investigations which are crucial for expanding our understanding of the time and hypoxic-dose dependent changes in appetite hormonal markers. Indeed, while up-to-date studies provided some evidence regarding both acute and prolonged effects of hypoxia on select markers the time course and kinetics (i.e., acclimatization effect) of these markers has yet to be investigated. Furthermore, potential differences in hormonal appetite modulation responses to repeated acute hypoxic exposures (e.g., few hypoxic sessions per week), as opposed to continuous prolonged exposures are currently unclear and worthy of a study. It is also important to note that hypobaric and normobaric hypoxia have previously been shown to differentially affect select cardiorespiratory and hematological parameters (Faiss et al., 2013). Accordingly, potential independent effect of hypobaria and hypoxia on hormonal appetite markers should be considered and further explored. Collectively, future well-controlled acute and prolonged studies are warranted to expand our understanding of hypoxia-induced hormonal appetite modulation and its kinetics in health and disease.

## Author contributions

TD collected the data, drafted and revised the manuscript and approved the final version.

### Conflict of interest statement

The author declares that the research was conducted in the absence of any commercial or financial relationships that could be construed as a potential conflict of interest.
